# A Prophecy

**Published:** 1886-10

**Authors:** 


### A PROPHECY.

THE ultimate result of the struggle in the American Medical Association will be the final extinction of the Medical Center.

That this was the necessary outcome of their course, would have been plainly evident to the leaders in the revolt, had they been capable of looking beyond their own immediate circles. Fifty years ago, the supremacy of the East, in matters medical, was perfectly natural. There were the only good schools of medicine then existing in America; the only development of clinical and other practical methods of instruction; even after medical colleges had arisen in the great cities of the West and South, the Eastern prestige was sustained by attracting to the old established schools the best of the teachers developed in other sections of the country. Hence, a professorship in the colleges of Philadelphia or New York was looked upon as the utmost pinnacle to which the ambitious surgeon could climb. The wise counsels which brought Gross to Philadelphia, and Marion Sims to New York, helped to perpetuate the supremacy of the East for years after the other sections of the country were fully abreast of her.

But a narrower policy began to prevail. The Eastern schools fell under the influence of rings and cliques of men, who sought their own elevation more than that of the institution upon which, leech-like, they had fastened themselves. Positions becoming vacated in the faculties were no longer filled by the best men who could be selected from the country at large, but were obtained through family or social influence, or else were conferred exclusively on graduates of the college itself. To such an extent was this exclusivism carried out, that out of a staff of *ninety-nine* in the teaching corps of an old Eastern university, only *two* were not her own graduates.

This short-sighted policy stimulated the growth of the Western colleges. As the prospect of Eastern professorships grew dim, the great teachers developed in other sections turned their attention to building up their own institutions; and, as neither money, enterprise, nor public spirit is wanting in any part of the country, their success was certain. While the Eastern schools have been emasculated by the system of "employing only cultured gentlemen to teach, because our students are gentlemen's sons," the other sections have been infiltrated with the most energetic and progressive men in the profession. *They* are the men who in swarms "cross the sea yearly to ransack the schools of Europe in search of the new and useful in medicine."

Still, the prestige of the East remained after all real superiority on her part had ceased to exist. All that was needed was a single rude blow to dissipate the delusion, and show the true relative strength of the two parties; and this blow she dealt herself. The exhibition of peurile spite, made by her alleged leaders, has brought on her the contempt of the whole American profession, except the few cads who rejoice, for once, in finding themselves in what they consider "good company." The harm done was not so much in what they did, as in the revelation it gave of the littleness and lack of foresight it showed in these "leaders." The time for such action to be dignified or useful has long passed. Achilles may sit sulking in his tent if he feels like it, but the fight will go on just the same. There are Ajax, Diomedes, Ulysses, and a host of nameless others, who are fully his equals in prowess, and his services will not be missed. In fact, he will find it difficult to obtain any place in the front rank among the men who have pressed on while he halted. New men will appear on the scene. Hereafter the Eastern colleges will have simply the standing which the quality of their work warrants. Their classes will dwindle to the mere personal following of the members of their faculties. Young and energetic men will push forward the schools which will share their honors. New institutions can with ease adopt high standards and equip themselves with late and improved appliances, of which the pecuniary interests of the older schools render them chary about availing themselves. Their text-books will be supplanted by those of more popular men; their authority will be disputed at every step; and, most unkindest cut of all, their consultation will be derived only from their own personal friends.

